# Factor analysis and subtyping significance of *CTNNB1* gene mutation detection in adamantinomatous craniopharyngioma

**DOI:** 10.1016/j.gendis.2023.101188

**Published:** 2023-11-30

**Authors:** Huarong Zhang, Chaohu Wang, Jun Fan, Rongrong Guo, Qianchao Zhu, Jun Pan, Junxiang Peng, Zhiyong Wu, Songtao Qi, Yi Liu

**Affiliations:** a Department of Neurosurgery, Institute of Brain Diseases, Nanfang Hospital, Southern Medical University, Guangzhou, Guangdong 510515, China; b The First School of Clinical Medicine, Southern Medical University, Guangzhou, Guangdong 510515, China; c The Laboratory for Precision Neurosurgery, Nanfang Hospital, Southern Medical University, Guangzhou, Guangdong 510515, China; d College of Traditional Chinese Medicine, Southern Medical University, Guangzhou, Guangdong 510515, China

Craniopharyngioma (CP) is a rare, histologically benign tumor located in the sellar region which is defined as a grade I tumor by the World Health Organization (WHO) classification.^1^ There are mainly two different clinicopathological subtypes of CP, the adamantinomatous CP (ACP) and the papillary CP (PCP).^1^ Although both variations have distinct histomorphological characteristics, an accurate diagnosis might be difficult to make, especially in tiny and/or fragmented specimens. Furthermore, there is a continuous scientific dispute about the occurrence of mixed forms and the cell of origin of these tumors.^2^ The *CTNNB1* gene mutation has been demonstrated to play a significant role in the tumorigenesis of ACP, and a growing body of research supported a high prevalence of *CTNNB1* mutation in ACP, while many studies have failed to detect the *CTNNB1* mutation in some ACP samples, thus resulting in a *CTNNB1* mutation rate of 16%–100%.^3^^,^^4^ Previously, work by Apps et al (2020) has demonstrated the high prevalence of *CTNNB1* mutations in ACP (100% in this study) when using a more sensitive TAm-seq sequencing method rather than Sanger sequencing.^5^ All the 22 ACP samples analyzed were found to carry the *CTNNB1* mutation by TAm-seq. A low mutant allelic frequency was found to correlate with the failure to detect the *CTNNB1* mutation by Sanger sequencing. Here, to figure out why the mutation rate of *CTNNB1* in ACP was inconsistent in the literature and to highlight the importance of the mutation for CP subtyping, Sanger sequencing was used to detect *CTNNB1* mutation in fresh-frozen tissues, formalin-fixed paraffin-embedded (FFPE) tissues, and primary ACP cells. Briefly, we observed that the *CTNNB1* mutation detection was influenced by the wet keratin/calcification, diaphragma sellae, and reactive glial tissue in ACP tissues. Hematoxylin and eosin (H&E) staining can be used to guide mutation identification to enhance the rate of *CTNNB1* mutation detection. An alternative for improving the mutation detection rate is to use primary ACP cells. Finally, The *CTNNB1* mutation is critical for CP subtyping, particularly for atypical CP.

To find the confounding variables that may influence the identification of *CTNNB1* mutation, a total of 43 ACPs with varying proportions of parenchyma was chosen to be sequenced (Table S1, 2). The results showed that the mutation rate of *CTNNB1* increased when the proportion of parenchyma was higher (Table S2). In addition, 18 samples with undetectable *CTNNB1* mutation were found to be rich with wet keratin/calcification, diaphragma sellae, and/or reactive glial tissue (Fig. 1A–C), which severely hampered the detection of *CTNNB1* mutation.

**Figure 1:**
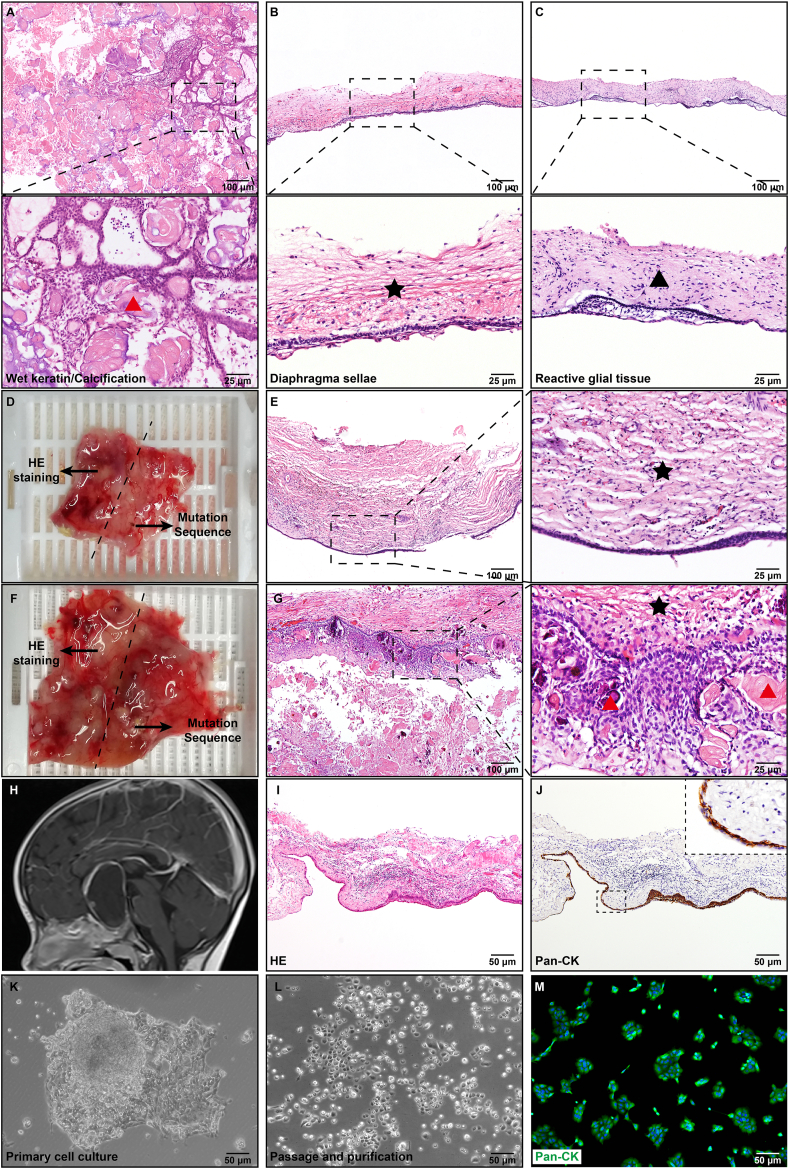
The factors affecting *CTNNB1* mutation detection in ACP tissue samples. **(A**–**C)** Hematoxylin and eosin (H&E) staining of ACP tissue sections and the representative images showing wet keratin/calcification (A), diaphragma sellae (B), and reactive glial tissue (C). Red arrowhead: wet keratin/calcification; black star: diaphragma sellae; black arrowhead: reactive glial tissue. The boxed area is enlarged and presented on the bottom. **(D, F)** Fresh surgical specimens were equally divided into two parts: half for *CTNNB1* mutation sequencing and the other half for histopathological verification. **(E)** H&E staining of ACP tissue sections and the representative images showing a case with extremely thick diaphragma sellae (black star) and negative *CTNNB1* mutation. **(G)** H&E staining of ACP tissue sections and the representative images showing a case with a high quantity of wet keratin/calcification (red arrowhead), diaphragma sellae (black star), and negative *CTNNB1* mutation. **(H)** Representative brain sagittal contrast-enhanced T1-weighted magnetic resonance (MR) image obtained from a patient with a cystic ACP before surgery. **(I, J)** The representative image of H&E staining and the representative image of Pan-CK staining showing a thin layer of ACP cells. **(K, L)** The representative reflection microscopy images showing the first generation of primary ACP cells around ACP tissue blocks (K) and primary ACP cells after passage and purification (L). **(M)** Immunofluorescence staining of primary ACP cells and the representative merged image of primary ACP cells treated with antibodies against Pan-CK (green) and DAPI (blue). ACP, adamantinomatous craniopharyngioma.

Fresh-frozen tissue, in addition to FFPE tissue, is also employed for ACP *CTNNB1* mutation sequencing. To further confirm the effects of the aforementioned confounding factors on *CTNNB1* mutation sequencing in fresh-frozen tissues, we selected fresh surgical specimens from seven ACP patients in our department and randomly divided them into two parts; half was stored at −80 °C in a refrigerator and used to detect *CTNNB1* mutation and the other half was used for subsequent histological verification (Fig. 1D, F). The final sequencing results revealed that five of the seven tissues submitted for *CTNNB1* mutation testing were positive and two were negative for the mutation (Tables S1, 3). The two tissues that were negative for *CTNNB1* mutation were identified by H&E staining, and the proportion of ACP parenchyma was about 20%, which was considered very low (Fig. 1E, G and Table S3); however, the proportion of ACP parenchyma in the five tissues that were positive for *CTNNB1* mutation was more than 50% (Table S3).

After removing the above confounding factors, to further identify whether the age of ACP FFPE tissue influenced the *CTNNB1* mutation, a total of 33 FFPE tissues of various ages was selected to be sequenced. The results showed that *CTNNB1* mutation was detectable in all tissue samples (Table S1, 4). Therefore, the age of FFPE tissue blocks did not affect the detection of *CTNNB1* mutation.

ACP is cystic in over 90% of cases and nearly 90% of ACPs show typically prominent calcifications (Fig. 1H). We know that utilizing FFPE or fresh-frozen tissues for cystic or calcified ACP mutation sequencing might result in negative findings, so we employed primary cell culture to purify ACP cells for *CTNNB1* mutant sequencing (Fig. 1I, J). The cultured primary ACP cells were polygonal or regular squares under the microscope, which is typical of epithelial cells (Fig. 1K, L). The epithelial marker pan-cytokeratin (Pan-CK) was identified by immunofluorescence staining after stable passage to three to five generations; the positive expression of Pan-CK indicated that the cultivated cells were ACP cells (Fig. 1M). A total of eight cases of primary ACP cells were cultured and identified, and the *CTNNB1* mutation was then detected. Simultaneously, we performed mutation sequencing on FFPE tissues from the corresponding patients. The results showed that the *CTNNB1* mutation was found in all eight cases of primary ACP cells, while the mutation was positive in only two FFPE tissues. Individual disparities in the mutation point remained (Table S1, 5). These results demonstrated that by culturing primary ACP cells, the *CTNNB1* mutation could be detected when the low content of ACP parenchyma in FFPE or fresh-frozen tissues resulted in negative *CTNNB1* mutation.

ACP is typically characterized by variable cystic, calcified, and solid components often surrounded by a florid glial and inflammatory reactive tissue. Epithelial whorls, palisading epithelium, stellate reticulum, and wet keratin/calcification are also present in typical ACP (Fig. S1A). Whereas PCP is typically composed of well-differentiated keratinized squamous epithelium (Fig. S1D). Therefore, it is enough for us to use H&E staining without immunohistochemistry to identify the typical ACP and PCP (Fig. S1B, C, E, F). However, CP is a histologically complex tumor whose pathology can be typical or atypical. There are two distinct manifestations of atypical ACP when the typical pathological features disappear. At this point, it is difficult to distinguish whether the pathological subtype is ACP or PCP only by H&E staining (Fig. S1G, J). To further clarify the pathological classification of CP, sequencing and immunohistochemistry were performed. Detectable *CTNNB1* mutation, undetectable BRAFV600E mutation, and positive expression of β-catenin in the cell nucleus are distinguishing features of ACP (Fig. S1H, I, K, L). Combined with the mutation sequencing and immunohistochemistry, the identification of these two subtypes will be more convincing.

In particular, the tumor tissues sometimes contain components similar to “wet keratin”, it is usually regarded as ACP and leads to misdiagnosis (Fig. S2A). Further immunohistochemistry and mutation sequencing can help us to precisely clarify that it is actually PCP (Fig. S2B–D). Furthermore, three CP cases exhibited not only palisading epithelium and stellate reticulum that are comparable to ACP (Fig. S2E) but also the features of keratinized squamous epithelium of PCP (Fig. S2I), which complicated our diagnosis. To address this problem, immunohistochemistry and mutation sequencing were further performed. The presence of *BRAF V600E* mutation, negative *CTNNB1* mutation, and the absence of nuclear aggregation of β-catenin suggested that these individuals should be classified as PCP (Fig. S2F–H, J–L).

In summary, we analyzed the *CTNNB1* mutation of ACP and found that the wet keratin/calcification, diaphragma sellae, and reactive glial tissue in ACP tissues influenced *CTNNB1* mutation detection. Then, to increase the rate of *CTNNB1* mutation detection, we offered two practical strategies. By excluding the aforementioned impact variables, *CTNNB1* mutation detection guided by H&E staining may be utilized to improve the rate of *CTNNB1* mutation detection, and another method is to employ cultured primary tumor cells. Further study revealed that the age of FFPE tissue blocks had no impact on ACP *CTNNB1* mutation detection. Finally, the *CTNNB1* mutation is of great significance in the classification of CP subtypes.

## Ethics declaration

All study procedures involving human participants were performed per the ethical standards of the Nanfang Hospital research committee and the 1964 Helsinki Declaration and its later amendments or comparable ethical standards.

## Author contributions

Huarong Zhang, Chaohu Wang, Jun Fan, Yi Liu, and Songtao Qi conceived and designed the experiments. Huarong Zhang, Chaohu Wang, and Qianchao Zhu performed the experiments. Jun Fan, Jun Pan, Junxiang Peng, and Yi Liu provided clinical specimens. Huarong Zhang, Chaohu Wang, Jun Pan, and Junxiang Peng analyzed the data. Huarong Zhang and Chaohu Wang wrote the paper. Huarong Zhang, Chaohu Wang, and Jun Fan contributed equally to this work. All authors read, helped to edit, and approved the final manuscript.

## Conflict of interests

The authors declare that they have no conflict of interests.

## Funding

This study was supported by grants from the National Natural Science Foundation of China (No. 82103033, 82204456, 82002646) and the President Foundation of Nanfang Hospital, Southern Medical University (No. 2022A002, 2022B024).

## References

[1] Müller H.L., Merchant T.E., Warmuth-Metz M., Martinez-Barbera J.P., Puget S. (2019). Craniopharyngioma. Nat Rev Dis Prim.

[2] Hölsken A., Sill M., Merkle J. (2016). Adamantinomatous and papillary craniopharyngiomas are characterized by distinct epigenomic as well as mutational and transcriptomic profiles. Acta Neuropathol Commun.

[3] Oikonomou E., Barreto D.C., Soares B., De Marco L., Buchfelder M., Adams E.F. (2005). Beta-catenin mutations in craniopharyngiomas and pituitary adenomas. J Neuro Oncol.

[4] Wang C.H., Qi S.T., Fan J. (2019). Identification of tumor stem-like cells in admanatimomatous craniopharyngioma and determination of these cells’ pathological significance. J Neurosurg.

[5] Apps J.R., Stache C., Gonzalez-Meljem J.M. (2020). CTNNB1 mutations are clonal in adamantinomatous craniopharyngioma. Neuropathol Appl Neurobiol.

