# Supplementary material for: Factor analysis and subtyping significance of CTNNB1 gene mutation detection in adamantinomatous craniopharyngioma
Source: Genes Dis. 2023 Nov 30;11(6):101188. doi: 10.1016/j.gendis.2023.101188 (PMC11320438; doi:10.1016/j.gendis.2023.101188)
Supplement: Multimedia component 1 [file mmc1.pdf]

Supplementary material for

Factor Analysis and Subtyping Significance of *CTNNB1* Gene Mutation Detection in Adamantinomatous Craniopharyngioma

Huarong Zhang[1,3,*], Chaohu Wang[1,3,*], Jun Fan[1,3,*], Rongrong Guo[1,2,3], Qianchao Zhu[1,3], Jun Pan[1,3], Junxiang Peng[1,3], Zhiyong Wu[4,#], Songtao Qi[1,3,#], Yi Liu[1,3,#]

Overview on Supplementary material:

- **Supplementary Figures**
  - Figure S1. **Histological, immunohistochemical, and molecular features of CP.**
  - Figure S2. **Histological, immunohistochemical, and molecular features of atypical PCP and "mixed-type CP".**

- **Supplementary Tables**
  - Table S1. **Summary of clinical data, mutational and immunohisto-chemical analyses in CP.**
  - Table S2. **The parenchyma proportion of ACP influenced the *CTNNB1* mutation detection.**
  - Table S3. **The *CTNNB1* mutation detection was influenced by the proportion of tumor parenchyma in seven fresh ACP frozen tissues.**
  - Table S4. **The age of ACP FFPE tissue blocks had no effect on *CTNNB1* mutation detection.**
  - Table S5. ***CTNNB1* mutation was identified in all primary cells in eight cases of ACP.**

- **Supplementary Materials and Methods**
  - Patient data
  - HE staining
  - Immunohistochemistry (IHC) staining
  - Primary cell culture
  - Immunofluorescence (IF) staining
  - DNA preparation
  - Mutational analysis

Supplementary Figures

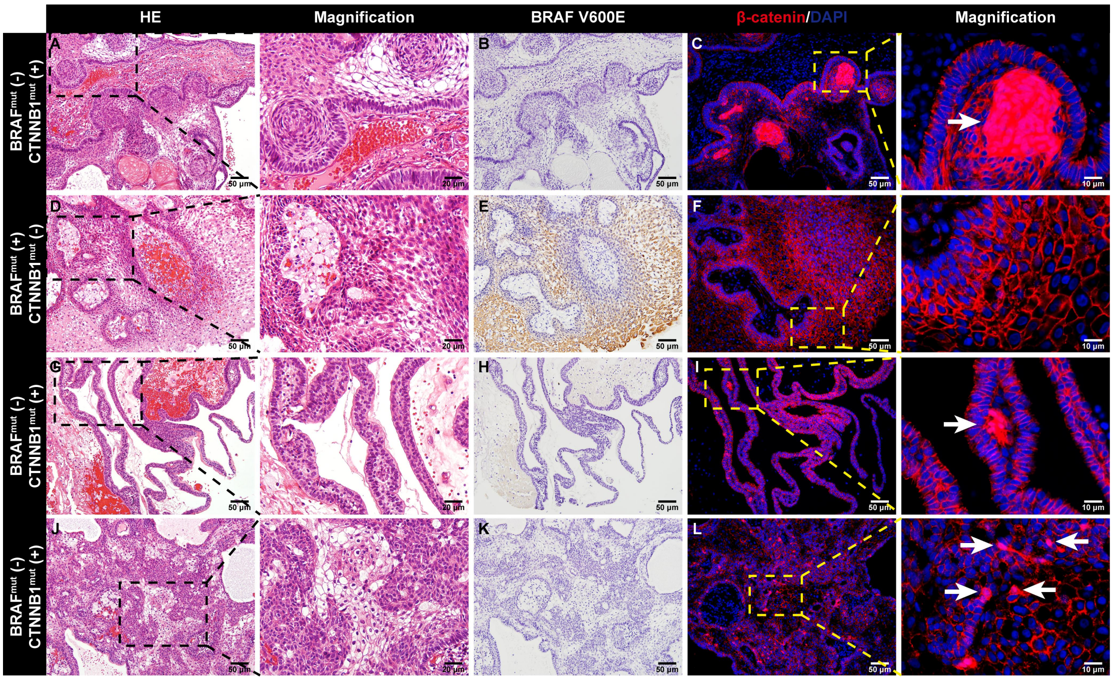

Figure S1. **Histological, immunohistochemical, and molecular features of CP.**

(**A**) H&E staining of typical ACP tissue sections. Representative images showing the classical features of ACP, such as EW, SR, PE, and wet keratin/calcification. Boxed area is enlarged and presented on the right. (**B**) Immumohistochemical staining of typical ACP tissue samples; representative images of BRAF V600E staining. (**C**) Immunofluorescence staining of typical ACP tissue samples, representative image of β-catenin staining shows β-catenin nuclear immunopositivity in EW (arrow). Boxed area is enlarged and presented on the right. (**D**) H&E staining of typical PCP tissue sections. Representative images showing the classical features of PCP, such as well-differentiated monomorphic squamous epithelium, fibrovascular, and capillary blood vessels. Boxed area is enlarged and presented on the right. (**E**) Immumohistochemical staining of typical PCP tissue samples; representative images of BRAF V600E staining. (**F**) Immunofluorescence staining of typical PCP tissue samples, representative image of β-catenin staining shows β-catenin membrane immunopositivity in all tumor cells. Boxed area is enlarged and presented on the right. (**G** and **J**) H&E staining of two atypical ACP tissue sections. Representative images showing the features of atypical ACP, such as atypical PE without typical SR and EW

(G), or the tumor cells arranged irregularly, and cells similar to basal cells of PCP in some areas with no typical PE, SR, EW, and wet keratin/calcification (J). Boxed area is enlarged and presented on the right, respectively. (**H** and **K**) Immumohistochemical staining of two atypical ACP tissue samples; representative images of BRAF V600E staining. (**I** and **L**) Immunofluorescence staining of two atypical ACP tissue samples, representative images of β-catenin staining shows β-catenin nuclear immunopositivity in scattered tumor cells (arrow). Boxed area is enlarged and presented on the right, respectively. $BRAF^{mut}$: BRAF mutation, $CTNNB1^{mut}$: *CTNNB1* mutation.

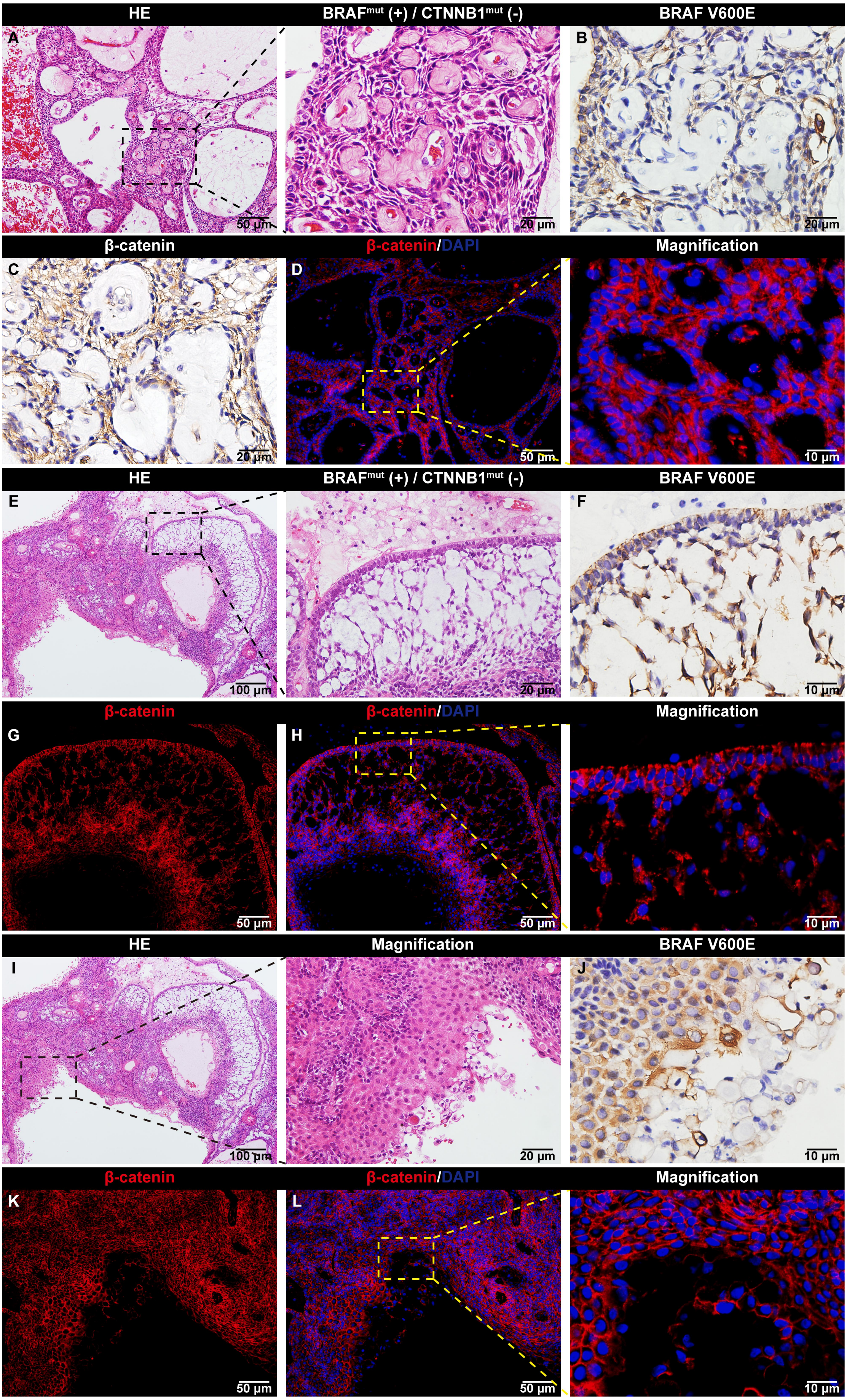
HE
BRAFmut (+) / CTNNB1mut (-)
BRAF V600E
A
B
50 μm
20 μm
20 μm
β-catenin
β-catenin/DAPI
Magnification
C
D
20 μm
50 μm
10 μm
HE
BRAFmut (+) / CTNNB1mut (-)
BRAF V600E
E
F
100 μm
20 μm
10 μm
β-catenin
β-catenin/DAPI
Magnification
G
H
50 μm
50 μm
10 μm
HE
Magnification
BRAF V600E
I
J
100 μm
20 μm
10 μm
β-catenin
β-catenin/DAPI
Magnification
K
L
50 μm
50 μm
10 μm

Figure S2. **Histological, immunohistochemical, and molecular features of atypical PCP and "mixed-type CP".**

(**A**) H&E staining of atypical PCP tissue sections. Representative images showing the features of atypical PCP with some components resemble wet keratin/calcification. Boxed area is enlarged and presented on the right. (**B-C**) Immumohistochemical staining of atypical PCP tissue samples; representative images of BRAF V600E staining (B) and β-catenin staining (C). (**D**) Immunofluorescence staining of atypical PCP tissue samples, representative image of β-catenin staining shows β-catenin membrane immunopositivity in all tumor cells. Boxed area is enlarged and presented on the right. (**E** and **I**) H&E staining of "mixed-type CP" tissue sections. Representative images showing the features of "mixed-type CP" with some cells resemble the PE and SR of typical ACP in some areas (E), and typical squamous epithelium can be seen in another area (I). Boxed area is enlarged and presented on the right, respectively. (**F** and **J**) Immumohistochemical staining of "mixed-type CP" tissue samples; representative images of BRAF V600E staining of different areas. (**G-H** and **K-L**) Immunofluorescence staining of "mixed-type CP" tissue samples, representative images of β-catenin staining show β-catenin membrane immunopositivity in all tumor cells. Boxed area is enlarged and presented on the right, respectively. BRAF^{mut}: BRAF mutation, $\mathrm{CTNNB1}^{mut}$: *CTNNB1* mutation.

Table S1. Summary of clinical data, mutational and immunohistochemical analyses in CP.

Case No.	Sex	Histology	CTNNB1 mutation sequencing			nuclear β-catenin	BRAFV600E	BRAFV600E-IHC	% tumor content	Age of FFPE tissue
			Fresh-frozen tissue	FFPE tissue	Primary cell					
1	M	ACP	/	WT	/	/	/	/	23.12	1.2
2	M	ACP	/	G34E	/	/	/	/	65.18	1.2
3	F	ACP	/	T41I	/	/	/	/	41.15	1.4
4	F	ACP	/	D32N	/	/	/	/	52.98	0.5
5	M	ACP	/	WT	/	/	/	/	10.02	0.3
6	F	ACP	/	WT	/	/	/	/	15.36	2.3
7	F	ACP	/	S33C	/	/	/	/	27.63	3.6
8	F	ACP	/	WT	/	/	/	/	9.85	4.1
9	M	ACP	/	D32N	/	/	/	/	85.43	0.1
10	M	ACP	/	WT	/	/	/	/	10.04	8.9
11	M	ACP	/	WT	/	/	/	/	19.22	7.6
12	M	ACP	/	WT	/	/	/	/	19.47	5.1
13	F	ACP	/	S33P	/	/	/	/	71.48	5.1

14	M	ACP	/	S37Y	/	/	/	/	75.53	4.8
15	F	ACP	/	WT	/	/	/	/	17.49	7.3
16	M	ACP	/	S37P	/	/	/	/	58.32	9.5
17	M	ACP	/	S33C	/	/	/	/	29.90	0.1
18	M	ACP	/	WT	/	/	/	/	34.28	3.3
19	M	ACP	/	WT	/	/	/	/	10.29	4.2
20	M	ACP	/	S33C	/	/	/	/	65.49	6.8
21	F	ACP	/	T41I	/	/	/	/	47.26	10.1
22	M	ACP	/	G34R	/	/	/	/	95.73	5.6
23	F	ACP	/	WT	/	/	/	/	4.47	6.3
24	M	ACP	/	WT	/	/	/	/	35.61	1.2
25	M	ACP	/	S37C	/	/	/	/	27.13	1.9
26	M	ACP	/	T41I	/	/	/	/	36.54	0.5
27	M	ACP	/	WT	/	/	/	/	8.38	0.7
28	F	ACP	/	T41I	/	/	/	/	24.06	1.2
29	F	ACP	/	S45P	/	/	/	/	85.33	1.7
30	F	ACP	/	WT	/	/	/	/	9.32	2.2

31	F	ACP	/	D32N	/	/	/	/	47.61	0.2
32	M	ACP	/	T41I	/	/	/	/	46.96	0.5
33	F	ACP	/	WT	/	/	/	/	14.74	0.8
34	M	ACP	/	WT	/	/	/	/	5.82	0.1
35	F	ACP	/	T41I	/	/	/	/	25.58	0.6
36	M	ACP	/	T41I	/	/	/	/	20.77	2.9
37	M	ACP	T41I	/	/	/	/	/	67.13	0.1
38	M	ACP	S37Y	/	/	/	/	/	55.48	0.1
39	M	ACP	G34R	/	/	/	/	/	59.32	0.3
40	F	ACP	T41S/F/I	/	/	/	/	/	63.02	0.5
41	F	ACP	D32G	/	/	/	/	/	77.49	0.5
42	M	ACP	WT	/	/	/	/	/	15.38	0.3
43	M	ACP	WT	/	/	/	/	/	20.14	0.2
44	M	ACP	/	D32G	/	/	/	/	90.85	12.1
45	F	ACP	/	T41I	/	/	/	/	81.63	10.1
46	M	ACP	/	D32G	/	/	/	/	55.94	13.2
47	F	ACP	/	S33C	/	/	/	/	68.78	10.7

48	M	ACP	/	D32N	/	/	/	/	70.42	11.6
49	M	ACP	/	S37Y	/	/	/	/	65.46	6.5
50	F	ACP	/	T41I	/	/	/	/	53.82	6.8
51	F	ACP	/	S33C	/	/	/	/	79.54	5.6
52	M	ACP	/	S45P	/	/	/	/	57.50	8.9
53	M	ACP	/	T41I	/	/	/	/	86.02	7.2
54	M	ACP	/	S37Y	/	/	/	/	62.81	9.2
55	F	ACP	/	G34E	/	/	/	/	50.09	5.1
56	F	ACP	/	D32N	/	/	/	/	52.43	4.9
57	M	ACP	/	S45P	/	/	/	/	73.36	4.2
58	M	ACP	/	T41I	/	/	/	/	50.41	4.8
59	M	ACP	/	S45P	/	/	/	/	69.97	3.1
60	M	ACP	/	S33C	/	/	/	/	55.38	3.5
61	F	ACP	/	D32G	/	/	/	/	54.74	3.9
62	M	ACP	/	G34E	/	/	/	/	60.98	1.2
63	F	ACP	/	S33P	/	/	/	/	78.70	2.3
64	M	ACP	/	G34E	/	/	/	/	58.87	1.4

65	M	ACP	/	T41I	/	/	/	/	57.39	1.4
66	F	ACP	/	S33C	/	/	/	/	91.08	2.3
67	M	ACP	/	S33C	/	/	/	/	72.83	2.4
68	F	ACP	/	D32G	/	/	/	/	65.78	1.3
69	M	ACP	/	G34E	/	/	/	/	58.10	1.5
70	F	ACP	/	S45P	/	/	/	/	55.59	1.5
71	M	ACP	/	T41I	/	/	/	/	54.69	1.8
72	M	ACP	/	S37Y	/	/	/	/	83.17	2.2
73	M	ACP	/	S33P	/	/	/	/	52.42	2.2
74	F	ACP	/	D32N	/	/	/	/	53.58	2.7
75	F	ACP	/	T41I	/	/	/	/	70.04	1.1
76	F	ACP	/	S33C	/	/	/	/	65.55	2.5
77	M	ACP	/	WT	G34R	/	/	/	9.82	1.8
78	M	ACP	/	WT	S37P	/	/	/	9.09	1.5
79	F	ACP	/	WT	D32N	/	/	/	12.06	1.5
80	F	ACP	/	WT	T41I	/	/	/	11.13	1.4
81	F	ACP	/	T41I	T41I	/	/	/	19.40	1.3

82	F	ACP	/	S33C	S33C	/	/	/	22.84	0.8
83	M	ACP	/	WT	S37C	/	/	/	15.88	0.8
84	F	ACP	/	WT	S33C	/	/	/	16.81	0.8
85	F	ACP	/	T41I	/	pos	WT	neg	91.07	5.8
86	M	ACP	/	T41I	/	pos	WT	neg	74.62	3.2
87	M	ACP	/	S33C	/	pos	WT	neg	54.98	4.5
88	F	ACP	/	D32N	/	pos	WT	neg	63.53	6.6
89	M	ACP	/	S37C	/	pos	WT	neg	57.16	2.9
90	M	ACP	/	T41I	/	pos	WT	neg	75.14	3.7
91	M	ACP	/	T41I	/	pos	WT	neg	40.67	1.8
92	F	ACP	/	S45P	/	pos	WT	neg	46.96	0.4
93	F	ACP*	/	S37Y	/	pos	WT	neg	82.95	2.3
94	M	ACP*	/	T41I	/	pos	WT	neg	94.51	3.3
95	F	ACP*	/	D32N	/	pos	WT	neg	83.42	0.5
96	F	ACP*	/	S33C	/	pos	WT	neg	73.76	0.7
97	M	PCP	/	WT	/	neg	mut	pos	/	2.4
98	M	PCP	/	WT	/	neg	mut	pos	/	2.2

99	M	PCP	/	WT	/	neg	mut	pos	/	1.8
100	M	PCP	/	WT	/	neg	mut	pos	/	2.3
101	M	PCP	/	WT	/	neg	mut	pos	/	0.8
102	M	PCP	/	WT	/	neg	mut	pos	/	0.2
103	M	PCP	/	WT	/	neg	mut	pos	/	0.9
104	M	PCP	/	WT	/	neg	mut	pos	/	1.4
105	F	PCP	/	WT	/	neg	mut	pos	/	2.5
106	M	PCP	/	WT	/	neg	mut	pos	/	3.2
107	M	PCP	/	WT	/	neg	mut	pos	/	0.1
108	F	PCP	/	WT	/	neg	mut	pos	/	1.5
109	M	PCP*	/	WT	/	neg	mut	pos	/	0.1
110	F	PCP*	/	WT	/	neg	mut	pos	/	0.8
111	M	PCP*	/	WT	/	neg	mut	pos	/	2.2

Nuclear staining pattern of β-catenin was analyzed immunohistochemically and classified as pos (positive) or neg (negative). ACP: adamantinomatous Craniopharyngioma; PCP: papillary Craniopharyngioma. FFPE tissue: formalin-fixed paraffin-embedded tissue; M: male; F: female; WT: wild type; mut: mutation. *=samples of patients diagnosed with mixed-type CP before mutational and immunohistochemical analyses. /=not detect.

Table S2. The parenchyma proportion of ACP influenced the *CTNNB1* mutation detection.

**Tumor parenchyma(%)**	＜30	30-50	＞50
**Sample cases**	22	7	14
**CTNNB1 mutation rate (%)**	27.3% (6/22)	71.4% (5/7)	100% (14/14)

Table S3. The *CTNNB1* mutation detection was influenced by the proportion of tumor parenchyma in seven fresh ACP frozen tissues.

**Case No.**	**Age**	**Sex**	**Diagnosis**	**Tumor parenchyma proportion(%)**	***CTNNB1* Sequencing**	
					**Mutation Site**	**Nucleobase Alternation**
1	20	M	ACP	67.13	T41I	ACC→ATC
2	7	M	ACP	55.48	S37Y	TCT→TAT
3	22	M	ACP	59.32	G34R	GGA→CGA
4	7	F	ACP	63.02	T41S/F/I	ACC→TTC
5	30	F	ACP	77.49	D32G	GAC→GGC
6	6	M	ACP	15.38	WT	—
7	4	M	ACP	20.14	WT	—

Table S4. The age of ACP FFPE tissue blocks had no effect on *CTNNB1* mutation detection.

**Age of FFPE blocks (years)**	1-3	3-5	5-10	≥10
**Sample cases**	15	6	7	5
***CTNNB1* mutation rate (%)**	100	100	100	100

Table S5. *CTNNB1* mutation was identified in all primary cells in eight cases of ACP.

**Case No.**	**Sex**	**Tissue for primary cell culture**	***CTNNB1* Sequencing**	
			**Mutation Site**	**Nucleobase Alternation**
1	M	ACP rich in diaphragma sellae	G34R	GGA→CGA
2	M	Calcified ACP tissue	S37P	TCT→CCT
3	F	ACP rich in nerve tissue	D32N	GAC→AAC
4	F	ACP rich in diaphragma sellae	T41I	ACC→ATC
5	F	ACP rich in diaphragma sellae	T41I	ACC→ATC
6	F	Calcified ACP tissue	S33C	TCT→TGT
7	M	Calcified ACP tissue	S37C	TCT→TGT
8	F	ACP rich in nerve tissue	S33C	TCT→TGT

SUPPLEMENTARY MATERIALS AND METHODS

Patient data: 111 patients with primary CP were investigated retrospectively in the Department of Neurosurgery of Southern Medical University from January 1, 2008, to October 1, 2019. All 111 patients were the first surgery, of which 96 patients were ACP and 15 patients were PCP. Among ACP, there were 56 children, defined as being younger than 18-years-old at diagnosis, (32 males and 24 females; age, 1-17 years) with a mean age of 7.36±4.26 years, and 40 adults (22 males and 18 females; age, 18-67 years) with a mean age of 36.90±15.20 years; PCP patients were all adults (12 males and 3 females; age, 19-56 years) with a mean age of 43.87±10.74 years. To ensure the freshness of the tissue, all specimens were obtained immediately after surgery, and written informed consent was obtained from each donor with the permission of the Institutional Review Board of Nanfang Hospital of Southern Medical University. This study was performed in accordance with the Declaration of Helsinki. The Research Ethics Committee at Nanfang Hospital of Southern Medical University gave its approval to all of the studies.

HE staining: HE staining was performed according to the routine protocol. Briefly, following deparaffinization and rehydration, 2 μm-thick sections were stained for 5 minutes with hematoxylin solution, dipped five times in 1% acid ethanol (1% HCl in 75% ethanol), and washed in distilled water. Sections were then stained for 3 minutes with eosin solution, dehydrated with graded alcohol, and cleared in xylene. Finally, using an Olympus BX51 microscope (Tokyo, Japan), mounted slides were viewed and photographed. The percentage of tumor parenchyma content (i.e. palisading epithelium, stellate reticulum and epithelial whorls) and non-tumor content (i.e. glial reactive tissue/nerve tissue, diaphragma sellae and wet keratin/calcification) was assessed histologically for each of the tumor samples. Tumor content was estimated as the proportion of tumor cell area within the whole area of the tumor section.

Immunohistochemistry (IHC) staining: The 2 μm-thick slices of FFPE tissues were

deparaffinized with xylene and hydrated using an ethanol gradient (70%–100%) after being maintained at 60°C for 2-4 hours in the oven. Endogenous peroxidase activity in tumor sections was blocked by incubation with 3% H2O2 for 10 minutes. Then, using 0.01 M citrate buffer (pH 6.0), heat-induced antigen retrieval was conducted in a microwave for 15 minutes. The slides were then rinsed with water and incubated overnight at 4°C in a refrigerator with BRAF V600E (1:1000; Spring Biosciences, Pleasanton, CA, USA) or β-catenin (ab32572; dilution 1:250; Abcam, Cambridge, MA). The slides were rinsed the next day and treated for 30 minutes with the corresponding secondary antibody (Beijing Biosynthesis Biotechnology Co. Ltd., Beijing, China), followed by 3, 3′-diaminobenzidine (DAB) (ZSGB-BIO) and hematoxylin staining. After that, an Olympus BX63 microscope (Tokyo, Japan) was used to analyze and photograph the slides.

Primary cell culture: Primary culture of ACP cells was carried out according to the established protocol as described previously[1]. In brief, the ACP specimens for primary culture were washed three times in phosphate-buffered saline (PBS) (Gibco, USA) before being sliced into 1 mm^3 fragments. According to the volume ratio of tumor size, 0.25% trypsin and Dulbecco's modified eagle medium (DMEM) at 1:1:2 were used to digest the cells for 40 minutes at 37°C. The cell suspension was neutralized using KGM™ Gold Keratinocyte Growth Medium (Lonza Ltd, CH-4002 Basel, Switzerland) after filtration. The cells were centrifuged, resuspended, and cultivated at 37°C in a humidified environment containing 5% CO_2. The filter residue was then smeared into the flask from the strainer and placed in the incubator without any reagents. KGM™ Gold Keratinocyte Growth Medium was added to the flask four hours later and cultured at 37°C in a humidified 5% CO_2 environment.

Immunofluorescence (IF) staining: The primary cells were fixed in 4% paraformaldehyde and permeabilized in PBS with 0.3% Triton X-100 for 15 minutes. The cells were blocked in the blocking solution for 1 hour after permeabilization (containing PBS with 10% goat serum). The cells were then treated with pan-CK

(ab7753; dilution 1:250; Abcam, Cambridge, MA) overnight at 4°C before being incubated with species-appropriate secondary antibodies (dilution 1:1000; Invitrogen) for 1 hour. Nuclei were stained with 4′, 6-diamidino-2-phenylindole (DAPI). Cells were then examined and photographed using an Olympus BX63 microscope (Tokyo, Japan). For tissue IF staining, after antigen retrieval was performed in a microwave for 15 minutes with 0.01 M citrate buffer (pH 6.0), the slides were blocked with 10% goat serum for 1 hour. The sections were then incubated overnight at 4°C with β-catenin (ab32572; dilution 1:250; Abcam, Cambridge, MA). The slides were rinsed the second day and treated for 1 hour with the corresponding secondary antibodies (dilution 1:1000; Invitrogen). DAPI was used to stain the nuclei. The slides were then photographed using an Olympus BX63 fluorescence microscope (Tokyo, Japan).

DNA preparation: Primary ACP cells of 1-3 generations, snap-frozen (−80°C) tissue samples, and FFPE tissue samples were employed for DNA extraction. To validate the tumor content, frozen sections of all tissue samples were microscopically examined. We collected as much DNA as possible from FFPE and frozen tissue. DNeasy Blood & Tissue Kit (Qiagen) and QIAamp DNA Mini Kit (Qiagen) were used to extract DNA from frozen tissue and FFPE tissue, respectively.The Maxwell system (Promega) was used to collected tumor genomic DNA from primary cells.

Mutational analysis: Polymerase chain reaction (PCR) amplification was performed using primers encompassing GSK-3β phosphorylation sites of the *CTNNB1*/β-catenin (exon 3) (primer sequence—forward: 5′-CTTGTGCGTACTGTCCTTCG-3′; reverse: 5′-ATTCTGGGCCATCTCTGCTT-3′) and kinase domain of *BRAF* (exon 15) gene (primer sequence—forward: 5′-TCATAATGCTTGCTCTGATAGGA-3′; reverse: 5′-GGCCAAAAATTTAATCAGTGGA-3′). PCR amplifications were performed in a 25 μl reaction mixture containing 100 ηg of tumor DNA, 2.5 μl of 10X PCR buffer, 0.8 μl of 10 mM dNTPs, 0.25 μl each of forward and reverse primers, and 0.8 μl of high-fidelity Taq polymerase (Applied Biosystems). Initial denaturation was performed for 5 minutes at 95°C, followed by 40 cycles of amplification consisting of

denaturation for 60 seconds at 95°C, annealing for 45 seconds at 60°C, and extension for 90 seconds at 72°C, with a final extension for 12 minutes at 72°C. Bidirectional sequencing was performed using the ABI 3730 sequencer (Applied Biosystems).

References

1. Chen M, Zheng SH, Liu Y, Shi J, Qi ST. Periostin activates pathways involved in epithelial-mesenchymal transition in adamantinomatous craniopharyngioma. J Neurol Sci 2016;360:49-54.
